# Islands in the Genome Promote Speciation

**DOI:** 10.1371/journal.pbio.0030318

**Published:** 2005-08-09

## Abstract

Using DNA microarrays, the authors identify three small regions of the genome that differ between two forms of hybridizing mosquitoes-regions that are likely to contain the genes responsible for reproductive isolation.

Have you ever wondered how the myriad insect forms—beetles, flies, dragonflies, mosquitoes, grasshoppers, ants, wasps, bees, and countless others—evolved? Insects make up 75% of all species known. The large number of insect species is probably a result of a combination of one or more factors: a high rate of formation of new species, or speciation, an ability to adapt to new environments and exploit new ecological niches, and a lower rate of extinction. Speciation, adaptation, and extinction are all controlled by the interplay between genetic and environmental factors. Understanding the genetic changes that lead to the formation of new species is an important area of research in evolutionary biology. [Fig pbio-0030318-g001]

**Figure pbio-0030318-g001:**
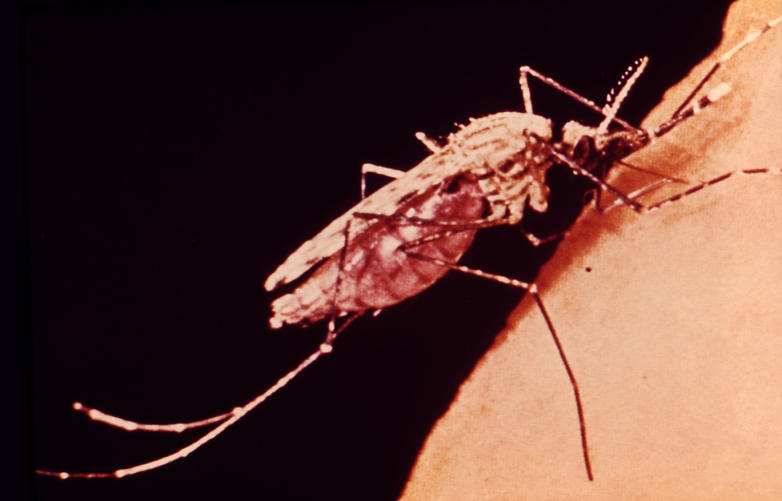
Two forms of the Anopheles mosquito, which transmits the malarial parasite, shed light on the genetic changes that prevent related species from producing fertile offspring— a condition of speciation. (CDC)

In a new study, Thomas Turner, Matthew Hahn, and Sergey Nuzhdin worked with the malaria mosquito Anopheles gambiae to uncover genes that may be driving speciation. A. gambiae exists in multiple forms that may be in the early stages of differentiating into separate species; on the other hand, they may be partially differentiated, co-existing races that could give us valuable information on genes responsible for racial differences in mosquitoes. Turner and colleagues focused on two forms, A. gambiae M and A. gambiae S, that sometimes mate and create hybrid forms in nature. While it's unclear whether the forms can produce fertile hybrid offspring in the wild, the progeny of lab matings appear to have no problems with fertility. This suggests that individuals either naturally prefer to mate with others of their own form, or that there must be environmental and/or genetic conditions that are not favorable for the survival of hybrid progeny in nature.

To study the genetic underpinnings of speciation, the researchers used DNA microarrays to identify global differences in the mosquito genomes. Using a combination of gene chips, statistics, and computational biology, Turner and colleagues found that the *M* and *S* genomes differ at just three regions. The researchers suggested that genes present here may be responsible for early speciation. These three “speciation islands” in the genome contain 67 predicted genes. In a preliminary analysis of seven of these genes, Turner and colleagues identified five that are different between the two Anopheles forms; these include genes that play a role in a range of cellular processes, including energy metabolism, response to sudden increases in temperature (heat shock), and ion transport across cell membranes. Future work focusing on the 67 genes hypothesized to reside in the divergent regions should yield interesting clues to the identity of genes that drive speciation, and the mechanism by which they do so.

This is a significant finding in the field of speciation research: in terms of methodology, this study shows that DNA microarrays can be used to identify regions of the genome that are different between two diverging species, allowing researchers to home in on potentially interesting genes. This study also shows that in spite of possible cross-flow of genetic material (natural hybrids between the two forms are found at a low frequency) between two populations, the populations can still be accumulating differences in their genomes—differences that could eventually lead to the formation of new species. Comparing results in Anopheles and the well-studied insect model Drosophila, in which scientists have also started identifying “speciation genes,” should tell us if similar genes are employed repeatedly in different genera during the formation of new species.

